# A Novel iRFP-Incorporated *in vivo* Murine Atherosclerosis Imaging System

**DOI:** 10.1038/s41598-018-32456-5

**Published:** 2018-09-28

**Authors:** Kaushalya Kulathunga, Michito Hamada, Yukiko Hiraishi, Mao Otake, Mai Thi Nhu Tran, Olivia Cheng, Junko Tanaka, Tomoki Sakasai, Shota Sakaguchi, Yuka Sugiyama, Bernd K. Fleischmann, Satoru Takahashi, Yoshihiro Miwa

**Affiliations:** 1 Department of Anatomy and Embryology, Faculty of Medicine, University of Tsukuba, 1-1-1, Tennodai, Tsukuba, Ibaraki 305–8575 Japan; 2 Ph.D. Program in Human Biology, School of Integrative and Global Majors, University of Tsukuba, 1-1-1, Tennodai, Tsukuba, Ibaraki 305–8575 Japan; 3 Laboratory Animal Resource Center, Faculty of Medicine, University of Tsukuba, 1-1-1, Tennodai, Tsukuba, Ibaraki 305–8575 Japan; 4 School of Medicine, Stony Brook University, Stony Brook, New York, 11794-8036 USA; 5 Institute of Physiology I, Life & Brain Center, Medical Faculty, University of Bonn, Sigmund-Freud-Str. 25, 53105 Bonn, Germany; 6 International Institute for Integrative Sleep Medicine (WPI-IIIS), University of Tsukuba, 1-1-1, Tennodai, Tsukuba, Ibaraki 305–8575 Japan

## Abstract

By using near-infrared fluorescent protein (iRFP)-expressing hematopoietic cells, we established a novel, quantitative, *in vivo*, noninvasive atherosclerosis imaging system. This murine atherosclerosis imaging approach targets macrophages expressing iRFP in plaques. Low-density lipoprotein receptor-deficient (*LDLR*^*−/−*^) mice transplanted with beta-actin promoter-derived iRFP transgenic (TG) mouse bone marrow (BM) cells (iRFP → *LDLR*^*−/−*^) were used. Atherosclerosis was induced by a nonfluorescent 1.25% cholesterol diet (HCD). Atherosclerosis was compared among the three differently induced mouse groups. iRFP → *LDLR*^*−/−*^ mice fed a normal diet (ND) and *LDLR*^*−/−*^ mice transplanted with wild-type (WT) BM cells were used as controls. The *in vivo* imaging system (IVIS) detected an enhanced iRFP signal in the thoracic aorta of HCD-fed iRFP → *LDLR*^*−/−*^ mice, whereas iRFP signals were not observed in the control mice. Time-course imaging showed a gradual increase in the signal area, which was correlated with atherosclerotic plaque progression. Oil red O (ORO) staining of aortas and histological analysis of plaques confirmed that the detected signal was strictly emitted from plaque-positive areas of the aorta. Our new murine atherosclerosis imaging system can noninvasively image atherosclerotic plaques in the aorta and generate longitudinal data, validating the ability of the system to monitor lesion progression.

## Introduction

Despite considerable therapeutic advances over the past 50 years, atherosclerosis-related cardiovascular diseases (CVD) remain the leading cause of death worldwide^1^. Conventional structural and functional imaging methods play increasingly important roles in better understanding such diseases and can be used to validate current therapeutic measures and to develop novel drug therapies^2^. Improved imaging technologies hold promise for accelerating drug development^1^.

Macrophages play essential roles in all phases of atherosclerosis, from the development of a fatty streak to processes that ultimately contribute to plaque rupture and myocardial infarction^3^. Leukocytes are central components of the inflammatory response, and plaque macrophages account for the majority of leukocytes in atherosclerotic plaques^4–6^. There is extensive evidence linking local macrophage infiltration with plaque characteristics and vulnerability^7^, and plaque macrophages have emerged as key imaging targets for inflammatory atheroma in animal models^8–12^. Consequently, techniques that can detect macrophages *in vivo* are useful for monitoring the development of atherosclerotic lesions. However, to our knowledge, there are few reports on direct methods that can be used to noninvasively measure the accumulation of macrophages without injecting imaging reagents. Therefore, user-friendly direct methods to noninvasively detect macrophage-rich plaques are in high demand in preclinical settings.

Bioluminescent proteins and visible light fluorescent proteins are powerful technologies that have been extensively used to further our understanding of complex processes. However, such proteins have limited utility *in vivo* because of their poor deep tissue penetration and high autofluorescence. In contrast, imaging with near-infrared fluorescence proteins within the 700–900 nm range of spectral wavelengths offers several advantages, including its high-sensitivity, nonionizing radiation and relatively simple operation^11,13^. A new near-infrared fluorescent protein (iRFP), a fluorescent mutant of *Rp*BphP2 bacteriophytochrome, was generated by Filonov *et al*.^12^. iRFP is a nontoxic, stable protein with excitation and emission wavelengths of 690 nm and 713 nm, respectively. iRFP is brighter, stronger and more stable than previous generations of similar fluorescent proteins^12^. These qualities make iRFP useful for *in vivo* imaging with great deep tissue penetration and minimal autofluorescence. Previously, Tran *et al*. generated transgenic (TG) iRFP mice with ubiquitous iRFP expression^14^. Because the expression of iRFP in hematopoietic cells was observed even in bone marrow-transplanted mice, we hypothesized that high iRFP fluorescence could be observed with the accumulation of macrophages within the atherosclerotic plaque area after transplantation of iRFP TG born marrow cells into X-ray-irradiated low-density lipoprotein receptor knockout (*LDLR*^*−/−*^) mice under hyperlipidemia conditions.

In this study, we established a noninvasive, *in vivo* atherosclerosis imaging system using iRFP hematopoietic cell-transplanted *LDLR*^*−/−*^ mice. To our knowledge, this is the first reported use of the endogenous iRFP fluorescence expression to image atherosclerotic lesions from 0 to 8 weeks without an invasive method or injection of imaging reagents. We believe that this novel noninvasive imaging approach will prove to be very helpful for monitoring disease progression in drug intervention studies with animal models.

## Results

### *In vitro* fluorescence expression of iRFP TG macrophages

**Figure 1:**
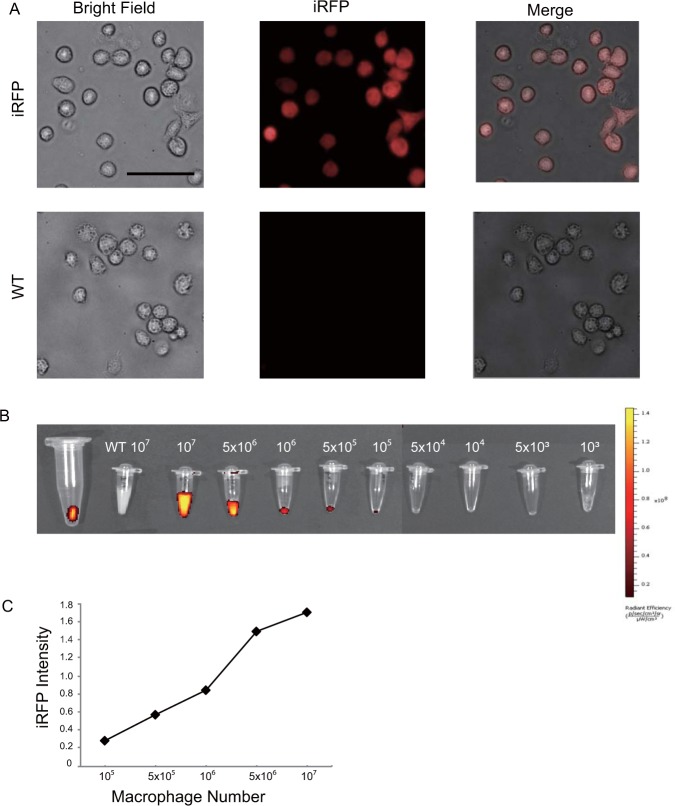
iRFP TG mouse macrophages express fluorescence *in vitro*. (**A**) Peritoneal macrophages from iRFP TG and WT mice observed by fluorescence microscope under a Cy5.5 filter. A clear, fluorescent signal was observed only in the iRFP TG macrophages (scale bar, 50 $\mu$m). (**B**) The observed iRFP signal with an augmenting number of iRFP TG mouse peritoneal macrophages in comparison to 1 × 10^7^ WT peritoneal macrophages by the IVIS spectrum under 710 nm and 760 nm excitation and emission wavelengths, respectively. The number of iRFP TG macrophage cells required to emit a detectable iRFP signal by the spectrum IVIS under the above conditions was determined to be 1 × 10^5^ cells. (**C**) The observed iRFP signal intensity with the corresponding macrophage number. A gradual increase in iRFP signal was observed with increasing cell number. The signal intensity was measured by the Living Image Software.

Initially, to validate the fluorescence intensity of iRFP TG mouse macrophages, we collected peritoneal macrophages from both iRFP TG and wild-type (WT) mice 3 days after injection of thioglycolate. Then, the cultured macrophages were observed by fluorescence microscope under a Cy5.5 filter. Clear, bright iRFP fluorescence signals were observed in the iRFP TG mouse macrophages, while no iRFP signal was observed in the WT macrophages (Fig. 1A). Next, we conducted an experiment to determine the minimum iRFP TG macrophage number that was required to produce a detectable fluorescent signal by IVIS in the *in vitro* conditions. iRFP TG peritoneal macrophages were collected in 0.2 ml tubes at amounts ranging from 1 × 10^3^ cells to 1 × 10^7^ cells. Clear fluorescence signals were detected even with 1 × 10^5^ iRFP TG macrophages, while no fluorescence signal was observed from the WT peritoneal macrophages, even at 1 × 10^7^ cells (Fig. 1B). The signal intensity increased exponentially with an augmented number of cells (Fig. 1C). The fluorescence signal intensity of 1 × 10^7^ iRFP TG macrophages was even higher than that of indocyanine green, which was used as a positive control. These results demonstrate that the iRFP TG macrophages display a bright iRFP signal and that the fluorescence intensity increased in a cell number-dependent manner. Moreover, the IVIS system was capable of capturing the cellular iRFP signal, and under our imaging conditions, a minimum of 1 × 10^5^ iRFP TG macrophages was required to detect a fluorescence signal *in vitro*.

### The iRFP signal is specific to atherosclerotic plaques

Because iRFP fluorescence is known to display excellent tissue penetration *in vivo* compared with its previous generations and other fluorescence proteins, we hypothesized that iRFP could be used for the *in vivo* imaging of atherosclerotic lesions. First, we transplanted bone marrow (BM) cells of iRFP TG mice into X-ray-irradiated *LDLR*^*−/−*^ mice, which were utilized as an inducible atherosclerosis model^15,16^. Two months after transplantation, the reconstitution efficiency of the hematopoietic system was verified by fluorescence-activated cell sorting (FACS) analysis of peripheral blood, and mice showing over 90% chimerism were selected for all further experiments.

**Figure 2:**
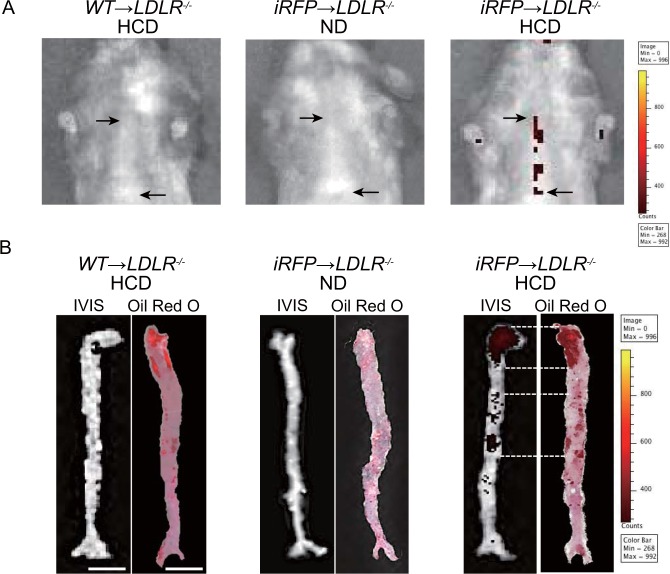
iRFP signal is expressed specifically in the plaques of HCD-fed iRFP → *LDLR−/−* mice. *In vivo* and *ex vivo* iRFP signals were identified by the IVIS under 710 nm and 760 nm excitation and emission wavelengths, respectively. The acquired IVIS images were adjusted to 268 (minimum) and 992 (maximum) counts of the color scale by Living Image software. (**A**) IVIS images of the thoracic area in HCD-fed WT → *LDLR*^*−/−*^, ND-fed iRFP → *LDLR*^*−/−*^ and HCD-fed iRFP → *LDLR*^*−/−*^. The thoracic signal (marked by arrows) was observed only in the HCD-fed iRFP → *LDLR*^*−/−*^ mice. (**B**) The comparison between *ex vivo* IVIS images and ORO staining of dissected aortas from the HCD-fed WT → *LDLR*^*−/−*^, ND-fed iRFP → *LDLR*^*−/−*^ fed and HCD-fed iRFP → *LDLR*^*−/−*^ mice. All imaging was performed after 8 weeks of feeding (scale bar, 5 mm). The data are from one representative experiment of at least two independent experiments.

Next, to induce atherosclerosis in iRFP → *LDLR*^*−/−*^ and WT → *LDLR*^*−/−*^ mice, we fed mice a specific high-cholesterol diet (HCD) that has a very low intensity of near-infrared autofluorescence. As a result, the IVIS analysis showed a clear iRFP fluorescence signal in the thoracic areas of iRFP → *LDLR*^*−/−*^ mice that were fed an HCD for 8 weeks (Fig. 2A, right panel). On the other hand, no thoracic iRFP fluorescence was observed in iRFP → *LDLR*^*−/−*^ mice that were fed a normal diet (ND) or in the HCD-fed WT → *LDLR*^*−/−*^ mice (Fig. 2A, middle and left panels). To confirm that the signal originated from the HCD-induced atherosclerotic plaques, we verified the iRFP signals in dissected aortas by the IVIS. Then, the aortas were stained with oil red O (ORO) to locate atherosclerosis plaque-positive areas. As expected, *ex vivo* iRFP signals and ORO-positive areas were observed in the aortas of the HCD-fed iRFP → *LDLR*^*−/−*^ mice. Moreover, the pattern of ORO staining seemed to very similar to the iRFP signal in the aorta (Fig. 2B, right panel). Importantly, we detected no iRFP signals in the aortas of the HCD-fed WT → *LDLR*^*−/−*^ mice or the ND-fed iRFP → *LDLR*^*−/−*^ mice. These results suggested that the iRFP signal was specifically emitted from the plaque area (Fig. 2B, left and middle panels). Because iRFP signals originate from hematopoietic cells from the BM, we speculated that the accumulation of primarily iRFP-baring macrophages and other immune cells in the plaques generated the signal. Altogether, these results suggest that our imaging system can capture murine atherosclerotic plaque areas *in vivo* in a noninvasive fashion.

### Fluorescence expression colocalizes with macrophage-rich plaques in iRFP-positive BM-transplanted mice

**Figure 3:**
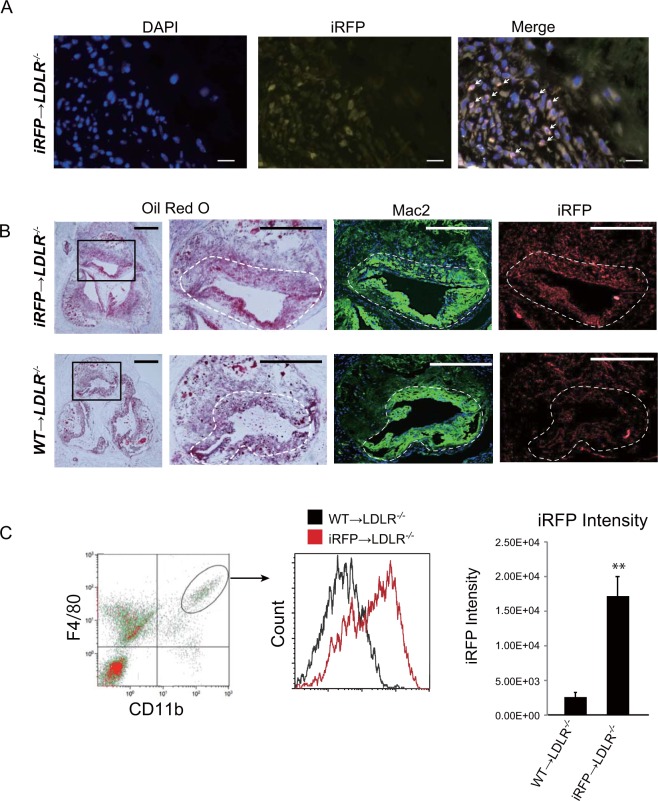
iRFP expressed in the atherosclerotic plaque macrophages is responsible for the plaque signal. (**A**) iRFP-positive cell accumulation was observed in the iRFP → *LDLR*^*−/−*^ mouse atherosclerosis plaques under an iRFP-specific 720 nm filter (red, scale bar, 20 $\mu$m). (**B**) Serial sections of atherosclerotic lesions around the aortic root of iRFP → *LDLR*^*−/−*^ and WT → *LDLR*^*−/−*^ mice fed the HCD for 8 weeks were stained with ORO and anti-Mac2 (macrophage marker, green), and the unstained adjacent section was observed under a Cy5.5 filter for iRFP expression (red; scale bar 500 µm for the first column and 100 µm for the rest). The macrophage-rich plaque area is marked by dotted lines. (**C**) The intensity of iRFP fluorescence expression in WT → *LDLR*^*−/−*^ and iRFP → *LDLR*^*−/−*^ plaque macrophages was assessed by flow cytometry after 8 weeks of atherosclerosis induction in digested mouse aortas. iRFP expression was measured in the CD11b and F4/80 double-positive cell fraction. The data are from one representative experiment of at least two independent experiments.

To unequivocally demonstrate that the observed fluorescence signals originated from macrophage-rich plaque areas, we carried out a histological analysis of the atherosclerotic plaques using 16–18-week-old iRFP → *LDLR*^*−/−*^ and WT → *LDLR*^*−/−*^ mice fed a HCD for 8 weeks. To determine whether iRFP expression could be observed after fixation in the plaques, unstained plaque sections were directly imaged under an iRFP-specific 720-nm filter (Fig. 3A). Accumulation of iRFP-positive cells (marked with arrows) was clearly observed in the iRFP → *LDLR*^*−/−*^ mouse atherosclerosis plaques. To determine whether the iRFP-expressing cells were plaque macrophages, consecutive sections of aortic root plaques were stained with ORO to localize the lipid-rich plaque areas and with an anti-Mac2 antibody to label the accumulated plaque macrophages. WT → *LDLR*^*−/−*^ mouse plaques were used as the negative control. As shown in Fig. 3B, a clear iRFP signal was observed in the Mac2-positive atherosclerotic lesions, as indicated by ORO staining in the iRFP → *LDLR*^*−/−*^ mice, whereas a negligibly low signal was observed in the WT-transplanted mouse plaques.

FACS analysis using adventitia-removed aortas can be used to identify immune cells of plaques^17^. We employed this FACS analysis to determine whether macrophages in the plaques express iRFP. CD11b and F4/80 double-positive cells (macrophages) were gated (Fig. 3C, left panel). The gating strategy and compensation control data are presented in Supplemental Fig. 1. As a result, the mean fluorescence intensity of the iRFP-incorporated plaque macrophages was approximately six-fold higher than that of the WT macrophages, whereas compared with the iRFP-expressing plaque macrophages, almost all WT macrophages showed a negligibly low level of autofluorescence (Fig. 3C, middle and right panels). These results indicated high colocalization of plaque macrophages and iRFP signals, implying that our imaging system specifically detects macrophage-filled plaques *in vivo*.

### The iRFP imaging system visualizes the macrophage-rich plaque burden *in vivo*

To our knowledge, few studies have been published that were able to image the plaque burden in animal models *in vivo*. Most of the time, *in vivo* imaging of atherosclerosis is limited to qualitative rather than quantitative imaging. We therefore assessed the ability of our imaging system to visualize differences in the macrophage-rich plaque burden *in vivo*. To induce different quantities of plaques, we adopted a strategy to feed the mice different amounts of the HCD, and consequently, the iRFP → *LDLR*^*−/−*^ mice were divided into three groups. One group was fed with the HCD for the entire experimental period and was called the “HCD” group. The second group was fed with the HCD and ND on alternating weeks and was named the “HCD/ND” group. The third group was fed only the ND, and thus, there was no atherosclerosis induction; this group was called the “ND” group. After 8 weeks of feeding, the mice in the three groups were imaged for atherosclerotic lesions. Then, the mice were sacrificed, and *ex vivo* imaging of the aortas was performed, followed by ORO staining.

**Figure 4:**
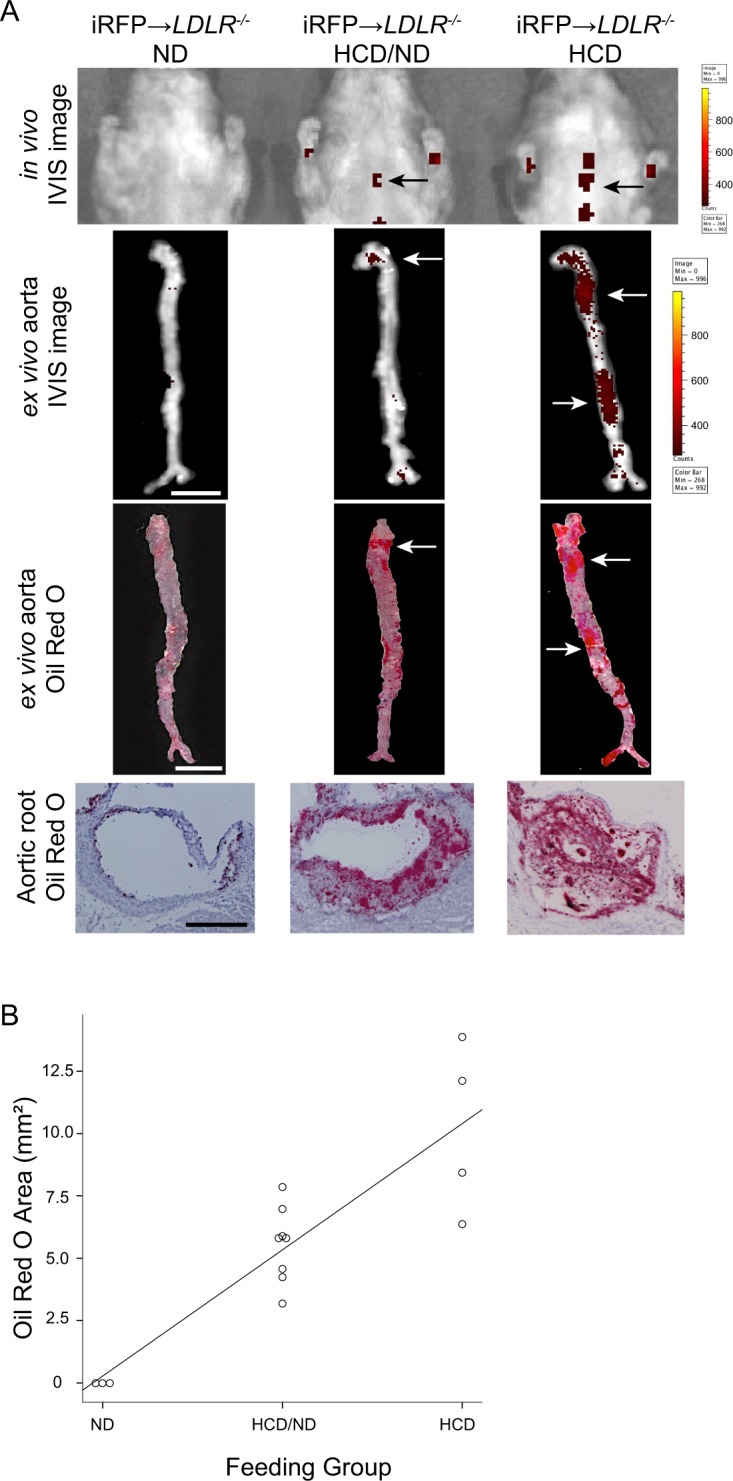
iRFP imaging system enables the observation of the differences in macrophage-rich plaque burden *in vivo*. The iRFP → *LDLR*^*−/−*^ mice were fed with different HCD quantities for 8 weeks to induce different atherosclerosis plaque burdens. The “ND group” was fed the ND for entire experimental period, the “HCD/ND group” was fed the HCD and ND on alternating weeks, and the “HCD group” was fed the HCD for the entire experimental period. (**A**) *In vivo* IVIS images, *ex vivo* aorta IVIS images (scale bar, 5 mm, signal-positive areas are marked by arrows), same aortas stained with ORO (scale bar, 5 mm, ORO-positive areas are marked by arrows), and aortic root sections stained with ORO (scale bar, 100 $\mu$m). (**B**) ORO-positive areas after 8 weeks of induction plotted against the feeding group. The ORO-stained areas showed a positive correlation with the feeding group at the *P* = 0.01 level. The data are from one representative experiment of at least two independent experiments.

As shown in Fig. 4A, the highest *in vivo* thoracic signals were clearly observed in the HCD group. Corresponding aortic *ex vivo* signal areas and ORO-positive areas could also be detected in this group. Negligible levels of *in vivo* fluorescent signals were observed in the ND group with no *ex vivo* aortic signals and ORO-negative staining. In the HCD/ND group, the observed signal area values were between those of the HCD and ND groups. ORO staining of the aortas and aortic valves showed that the different HCD feeding patterns induced different extents of atherosclerosis in each group, and the observed *in vivo* iRFP signal areas demonstrated a signal distribution corresponding to the actual plaque-positive areas. Aortic ORO-positive areas were significantly correlated with the amount of HCD that was fed to the mice, i.e., induction percentage (Spearman’s rank correlation = 0.865, (ND) group: n = 3, (HCD/ND) group: n = 8, (HCD) group: n = 4, P = 0.01), confirming that the different plaque burdens resulted from differences in atherosclerosis induction by the HCD (Fig. 4B).

**Figure 5:**
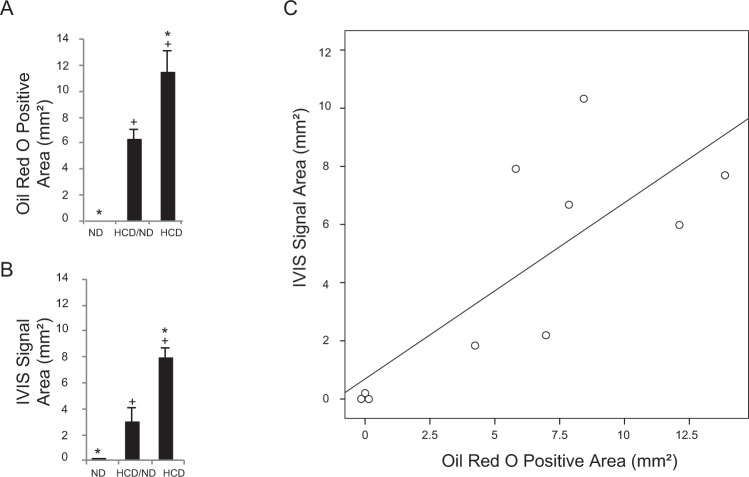
Atherosclerosis lesion areas are closely related to the *in vivo* iRFP signal areas. (**A**) ORO-positive areas and (**B**) *in vivo* IVIS signal areas in all three groups were measured by ImageJ software. *P < 0.05 versus the “ND group”, ^+^*P* < 0.05 versus the “HCD/ND group” (Student’s t-test). **(C**) A significant positive correlation was observed between the ORO-positive areas and the *in vivo* fluorescence signal areas at the level of *P* = 0.01. The data are from one representative experiment of at least two independent experiments. Quantified data are presented as the mean ± s.e.m.

As shown in Fig. 4A, the ORO-positive areas and *in vivo* iRFP signal areas were not identical, but closely related. We quantified the actual plaque area in all groups by measuring the ORO-positive areas (Fig. 5A). *In vivo* iRFP signal-positive areas were also quantified (Fig. 5B). Notably, a significant correlation was observed between the iRFP *in vivo* signal area and the *ex vivo* ORO-positive plaque area of the aortas (Spearman’s rank correlation = 0.782, n = 10, P = 0.01, Fig. 5C). Collectively, these results suggest that our iRFP-based imaging system can be used to visualize macrophage-rich plaque burden *in vivo* via IVIS, taking advantage of the thoracic iRFP fluorescence signal without any invasive procedures.

### iRFP fluorescence is related to plaque progression

**Figure 6:**
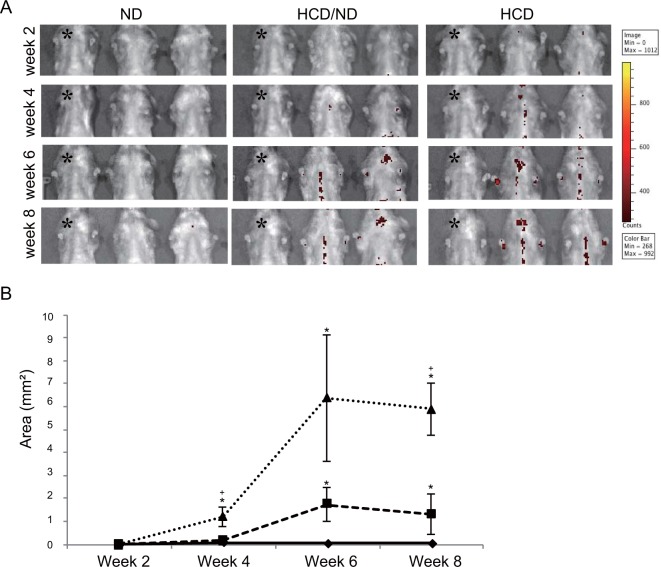
iRFP imaging system enables to the observation of the time course of plaque progression *in vivo*. (**A**) Thoracic IVIS images of mice in all three groups were acquired every 2 weeks. The ND-fed iRFP → *LDLR*^*−/−*^ mice were used as a negative control in each imaging session and are denoted by * here. (**B**) The mean signal area of each group is plotted from week 2 to week 8. (triangle, “HCD group”; square, “HCD/ND group”; diamond; “ND group”). The data are from one representative experiment of at least two independent experiments. Quantified data are presented as the mean ± s.e.m. *P < 0.05 versus the ND group, ^+^*P* < 0.05 versus the HCD/ND group (Student’s t-test).

To continuously monitor iRFP fluorescence, IVIS images were acquired every two weeks to observe the thoracic signal in the three groups fed different amounts of HCD. Figure 6A shows the disease progression imaging up to 8 weeks after atherosclerosis induction. We used ND-fed iRFP → *LDLR*^*−/−*^ mice as the negative control in each imaging session (denoted by * in Fig. 6A). From the fourth week of induction, we observed thoracic signals in the HCD/ND and HCD groups, whereas only negligible signals were detected in the ND group. The thoracic iRFP signals were clearly observed in the same mice at week 6 and week 8 after induction. As expected, the HCD group mice showed the highest signal area, followed by the HCD/ND group mice (Fig. 6A). Moreover, we quantified the signal area from weeks 2 to 8 in the three groups. These data showed that the area of the iRFP signals clearly differed among the HCD group, the HCD/ND group, and the ND group. Overall, these results indicate that our imaging system can clearly capture the time course of disease progression in individual mice in a relatively straightforward fashion. This system also allows differences in plaque burden to be quantified among mice in the same group.

## Discussion

For the first time, we showed here that endogenous iRFP fluorescence as a sensitive marker for imaging deep tissue pathologies, such as aortic atherosclerotic lesions. Previously, Min Htun *et al*. took advantage of the near-infrared autofluorescence emitted by intraplaque hemorrhage to image vulnerable plaques in carotid arteries *in vivo*^18^. Though this approach has greatly advanced, it still possesses some limitations, as it can only image hemorrhagic plaques and may have limited ability for deep tissue imaging due to its dependence on autofluorescence. Here, we demonstrate the advanced use of endogenous iRFP fluorescence expression in macrophages to visualize plaque development in deep tissues, such as the thoracic aorta.

The accumulation of iRFP-incorporated macrophages and other immune cells in plaques produces a signal that is strong enough to be detected by the IVIS and to distinguish lesions from normal tissue. Recent reports show that while monocytes/macrophages play a major role in plaque development, the infiltration of other cells, such as neutrophils, dendritic cells, B cells and T cells, is also responsible for plaque development^19^. On the other hand, as white blood cells and erythrocyte express iRFP in iRFP TG, iRFP signals were detected in capillary-dense regions with no hair, such as the paws and the mandible. These signals may be caused by either minor fluorescence of red blood cells in capillary beds or increased white blood cells derived from inflammation after HCD (Figs 2A, 4A and 6A)^14^. However, our FACS analysis data showed that iRFP was predominantly expressed in the F4/80^+^CD11b^+^ plaque macrophage population, whereas a very small population of F4/80^−^CD11b^−^cells express iRFP at negligible intensities (data not shown). Based on these data, it is reasonable to consider that the majority of iRFP fluorescence in atherosclerotic plaque originates from macrophages.

As monocyte infiltration is the initial event in plaque development^20,21^, incorporating iRFP into the monocyte lineage cells enables plaque detection and the observation of plaque-prone areas from a very early stage. Moreover, it has been shown that infiltrated macrophages can locally proliferate to increase their number in the lesion, and self-proliferation dominates in the advanced stages^22,23^. In our BMT system, iRFP-expressing macrophages from the transplanted hematopoietic system infiltrate the initial lesion and initiate a signal. In the event of local proliferation of infiltrated macrophages, our system benefits from the fact that locally proliferated macrophages also express iRFP fluorescence and contribute to the signal, as iRFP is genetically incorporated and can be genetically carried to subsequent generations. Thus, a continuous signal is ensured throughout plaque progression.

Continuous endogenous fluorescent expression enables time course monitoring of plaques with real-time longitudinal data generated by the same mice. This is greatly beneficial, as it reduces the number of research animals used in a study and facilitates data interpretation, as experimental variations are less. Furthermore, long-term *in vivo* observation allows individual differences in disease patterns and drug therapy to be studied. As an example, it was reported that the aortic root, the lower curvature of the aortic arch, and branch points of the aortic arch are frequent predilection sites for initial plaque development^24^. Though an initial signal is expected to be visible in the aortic arch, we found that some mice also showed initial signal development in the descending aorta, which may due to individual differences in mice with slight differences in predilection sites. In this scenario, our imaging system has the advantage allowing plaque formation to be observed in real time. This imaging system can be used to observe the general patterns and individual differences in predilection sites for atherosclerosis in the thoracic aorta.

Most recently, Calcagno *et al*. successfully demonstrated integrated ^18^F-FDG PET and a dynamic contrast-enhanced (DCE) MRI imaging protocol for quantification of several plaque parameters, including plaque inflammation and plaque burden, in rabbits^25^. Though the clinical use of this method is promising, its requirements of highly skilled professionals, time, and frequent injections of radioactive imaging agents could be unfavorable for preclinical animal study settings. Here, we successfully showed that our new imaging system has the capability to generate *in vivo* data on macrophage-rich plaque burden via a comparatively simple and feasible approach. However, it must be noted that the observed signal is from plaque macrophages and may not represent the whole plaque burden depending on the stage of atherosclerosis. Early lesions are rich in macrophages, and severe lesions can show partly noncellular areas. Therefore, the macrophage-rich plaque area may closely represent the whole plaque in the early stages, as shown in our 8-week study, but could be a limitation in interpreting the burden in advance plaques.

The significant positive correlation observed between the ORO-stained area and the *in vivo* IVIS signal area is advantageous for commenting on the actual plaque area based on the IVIS signal. However, the observed correlation coefficient seems slightly low, which could be explained by the fact that ORO stains both the foam cell lipid accumulations and extracellular lipid deposits, while the fluorescence signal is from macrophage foam cells. Moreover, a cut and opened aorta was used in *ex vivo* ORO staining, while *ex vivo* IVIS images were taken before cutting and opening the aorta. This can also contribute to configuration changes and may lead to differences in the plaque area measurements between the two conditions.

In human atherosclerosis, plaque rupture and subsequent thrombosis are the main underlying cause of acute cardiovascular outcomes varying from unstable angina to sudden death^26^. It has been reported that plaque vulnerability increases with the plaque macrophage population^7,27–29^. Here, we showed that the number of iRFP TG macrophages relates to the iRFP signal intensity. Therefore, with a proper translational approach, we believe that iRFP-based imaging systems could be developed to predict human atherosclerosis plaque vulnerability *in vivo*.

From a practical, preclinical point of view, our system is less invasive and less hazardous than other existing models that require the injection of imaging fluorescent/radioactive dyes at every imaging session. This system can be easily modified to acquire greater sensitivity and specificity by using a more sensitive detection system, such as photoacoustic imaging. In addition, greater signal specificity on the biological side could be achieved by limiting iRFP expression specifically in macrophages or, more specifically, in atherosclerotic plaque macrophages. Using a macrophage-specific “cre” driver or specifically targeting upregulated genes during plaque foam cell formation to incorporate iRFP may be successful approaches. Studies evaluating further advancements of the system are underway.

In conclusion, a method for visualizing atherosclerotic lesions by using iRFP fluorescence was developed. The atherosclerotic lesion areas of at least 5 mice were evaluated via a one-time detection approach by using an IVIS system noninvasively and without any injection reagent. Therefore, this method may be a good system for drug discovery or for easily determining the atherosclerosis phenotype of gene-targeting mice.

## Methods

### Mouse model generation

We used iRFP TG mice that ubiquitously expressed iRFP by using a beta-actin promoter^14^. BM cells were collected from iRFP TG mice. BM cells (1 × 10^7^) were transplanted into 10–12-week-old, lethally X-ray-irradiated (7 Gy) female *LDLR*^*−/−*^ mice by tail vein injection. Eight weeks after transplantation, establishment of the transplanted hematopoietic systems in the recipient mice was confirmed by testing the peripheral blood chimerism using iRFP fluorescence. Mice with chimeras higher than 90% were used for further experiments. WT BM cell-transplanted *LDLR*^*−/−*^ mice were used as a negative control. Moreover, reconstituted mouse blood parameters were checked before inducing atherosclerosis. The iRFP-expressing BM-transplanted mice are abbreviated as iRFP → *LDLR*^*−/−*^, and the WT mice, as WT → *LDLR*^*−/−*^. The mice were maintained under specific pathogen-free conditions in a laboratory animal resource center at the University of Tsukuba. All experiments were performed in compliance with relevant Japanese and institutional laws and guidelines and were approved by the University of Tsukuba animal ethics committee (authorization number 17–156).

### Chimerism analysis

Approximately 300 µl of blood was collected in EDTA-coated tubes from the transplanted mice by facial venous puncture. The cells were prepared according to a previously published method^14^. The cells were stained with antigen-presenting cell (APC)-conjugated anti-mouse CD 45 antibody (BioLegend, USA) for 30 min, washed, and suspended in phosphate-buffered saline (PBS) for analysis with a Gallios flow cytometer (Beckman coulter, USA). iRFP fluorescence was detected by the FL7 (725/20) channel. Chimerism was determined by the percentage of cells that were positive for both iRFP and CD 45.

### Atherosclerosis induction and *in vivo* imaging

Eight weeks after transplantation, atherosclerosis development was initiated by feeding the mice an atherogenic HCD with 1.25% cholesterol (Oriental Yeast Co. Ltd, Japan). The diet was especially designed to express no fluorescence (HCD). Control groups were fed with a made-to-order nonfluorescent normal diet (NF-ND). The live *in vivo* imaging system (IVIS; Perkin Elmer, USA) was used as the imaging device. Live imaging was conducted from day 0 of atherosclerosis induction and monitored every 2 weeks. Mice were anesthetized by inhalation anesthesia (Perkin Elmer, USA) with isoflurane for induction and anesthesia maintenance during imaging. The ventral surface of the body was shaved and subjected to imaging. All the IVIS images were acquired with excitation/emission wavelengths of 625/720 nm and 710/760 nm and with an exposure time of 1 second (1 s).

### Atherosclerotic area calculation

Acquired IVIS images were adjusted to the same minimum and maximum values of the color scale by Living Image Software (Perkin Elmer, USA) for comparison. The values of the negative controls, which did not show any autofluorescence, were selected and set as the minimum (268 counts) and maximum (992 counts) values. All the images were normalized to the selected values. In the present study, we were only focused on imaging atherosclerotic lesions in the thoracic aorta. The region of interest (ROI) was manually traced via Living Image Software. Photoshop software (Adobe System, USA) was used to separate the ROIs. The specific signal area was measured by the edge detection function of ImageJ software (National Institutes of Health, USA).

### Aortic digestion and plaque cell FACS analysis

After 8 weeks of atherosclerosis induction, the iRFP → *LDLR*^*−/−*^ and WT → *LDLR*^*−/−*^ mice were sacrificed by CO_2_ inhalation. Sacrificed mice were fully infused with a slow injection of 20 ml of PBS. Four percent paraformaldehyde (PFA) was not used for the perfusion. The aortas were carefully dissected and cleaned. Aorta digestion and collection of a single-cell suspension were performed as previously published^17^. The collected cells were washed with PBS and incubated with a fluorescein isothiocyanate (FITC)-conjugated anti-mouse F4/80 antibody (Bio-Rad, USA) and a phycoerythrin (PE)-conjugated anti-mouse CD11b antibody (Biolegend, USA) on ice for 30 min. Cells were washed and resuspended in PBS. FACS analysis was carried out using a Gallios flow cytometer (Beckman coulter, USA). Initially, F4/80-positive, CD11b-positive cells (macrophages) were gated, and iRFP expression of the double-positive macrophages was detected by the FL7 (725/20) channel. The FACS results were analyzed by Kaluza software (Beckman coulter, USA) and FlowJo software (FlowJo LLC, USA).

### *Ex vivo* macroscopic analysis of the aortic plaque area

We sacrificed mice after 8 weeks of acquiring IVIS images. The aortas were carefully dissected and cleaned after slow perfusion with PBS. *Ex vivo* IVIS images of each aorta were acquired shortly after dissection (excitation/emission of 710/760 nm). We followed a previous method to identify the aortic plaque areas by ORO (Wako, Japan) staining^30^. Briefly, after further cleaning and removal of the adventitia, the aortas were fixed overnight in 4% PFA. The aortas were longitudinally opened and stained with ORO. Immediately after staining, opened aortas were carefully mounted on a black paper and photographed. ORO-positive areas were measured by analyzing the images via Photoshop and ImageJ software.

### Histological analysis of atherosclerotic plaques

For the histological analysis of atherosclerosis plaques, dissected hearts were fixed in 4% PFA for 4–5 hours and incubated in 30% sucrose at 4 °C overnight. Fixed hearts were horizontally cut, and the half containing the apex was frozen in optimal cutting temperature (O.T.C.) embedding medium (Sakura Finetek, Japan). Serial sectioning was performed in the area of the aortic root by cryotome (Leica, Germany). Each section was 6 µm thick. Hematoxylin and eosin staining and ORO staining were performed as described in established protocols^3,14^. For immunohistochemical analysis of Mac2 expression in the plaque macrophages, frozen sections were incubated with a 1:200 dilution of rat anti-mouse Mac2 antibody (Cedarlane, Canada). For secondary fluorescent staining, a 1:500 dilution of Alexa Fluor 488-conjugated chicken anti-rat IgG secondary antibody was used (Invitrogen, USA). Stained histological sections were observed with a BioRevo fluorescence microscope (Keyence, Japan). iRFP fluorescence expression was observed in consecutive, unstained sections under a Cy5.5 filter using a fluorescence microscope (Olympus, Japan).

### Assessing macrophage iRFP expression and intensity

Healthy 2–3-month-old iRFP TG and WT B6 mice were intraperitoneally injected with 3 ml of thioglycolate (Becton Dickinson, USA). Three days after the injection, the mice were sacrificed, and the accumulated peritoneal macrophages were collected in PBS. Serial dilutions of iRFP TG macrophages were made ranging from 1 × 10^3^ cells/sample to 1 × 10^7^ cells/sample. The dilutions were centrifuged, and cell pellets were obtained. The fluorescence of the samples was imaged by the IVIS under 710 nm and 760 nm excitation and emission wavelengths, respectively. WT macrophages (1 × 10^7^) were used as a negative control. The fluorescence intensities of different numbers of cells were measured by the Living Image Software (PerkinElmer/Caliper, USA) and plotted against the cell number. Another sample of collected iRFP and WT macrophages were washed with PBS, and 1 × 10^6^ cells were cultured for more than one hour at 37 °C with DMEM (Life Technologies, USA) containing 10% fetal bovine serum (FBS) (Sigma-Aldrich, USA) and 1% penicillin. After incubation, the used medium was carefully removed with floating cells, and the adhered macrophages were isolated. iRFP expression in the cultured cells were observed under a Cy5.5 filter by a fluorescence microscope. (Olympus, Japan).

## Electronic supplementary material

Supplemental figure1
