# Supplementary material for: A Novel iRFP-Incorporated in vivo Murine Atherosclerosis Imaging System
Source: Sci Rep. 2018 Sep 28;8:14515. doi: 10.1038/s41598-018-32456-5 (PMC6162307; doi:10.1038/s41598-018-32456-5)
Supplement: Supplementary file 1 — Supplemental figure1 [file 41598_2018_32456_MOESM1_ESM.pdf]

A Novel iRFP-Incorporated *in vivo* Murine Atherosclerosis Imaging System

Kaushalya Kulathunga, Michito Hamada, Yukiko Hiraishi, Mao Otake, Mai Thi Nhu Tran, Olivia Cheng, Junko Tanaka, Tomoki Sakasai, Shota Sakaguchi, Yuka Sugiyama, Bernd K. Fleischmann, Satoru Takahashi and Yoshihiro Miwa

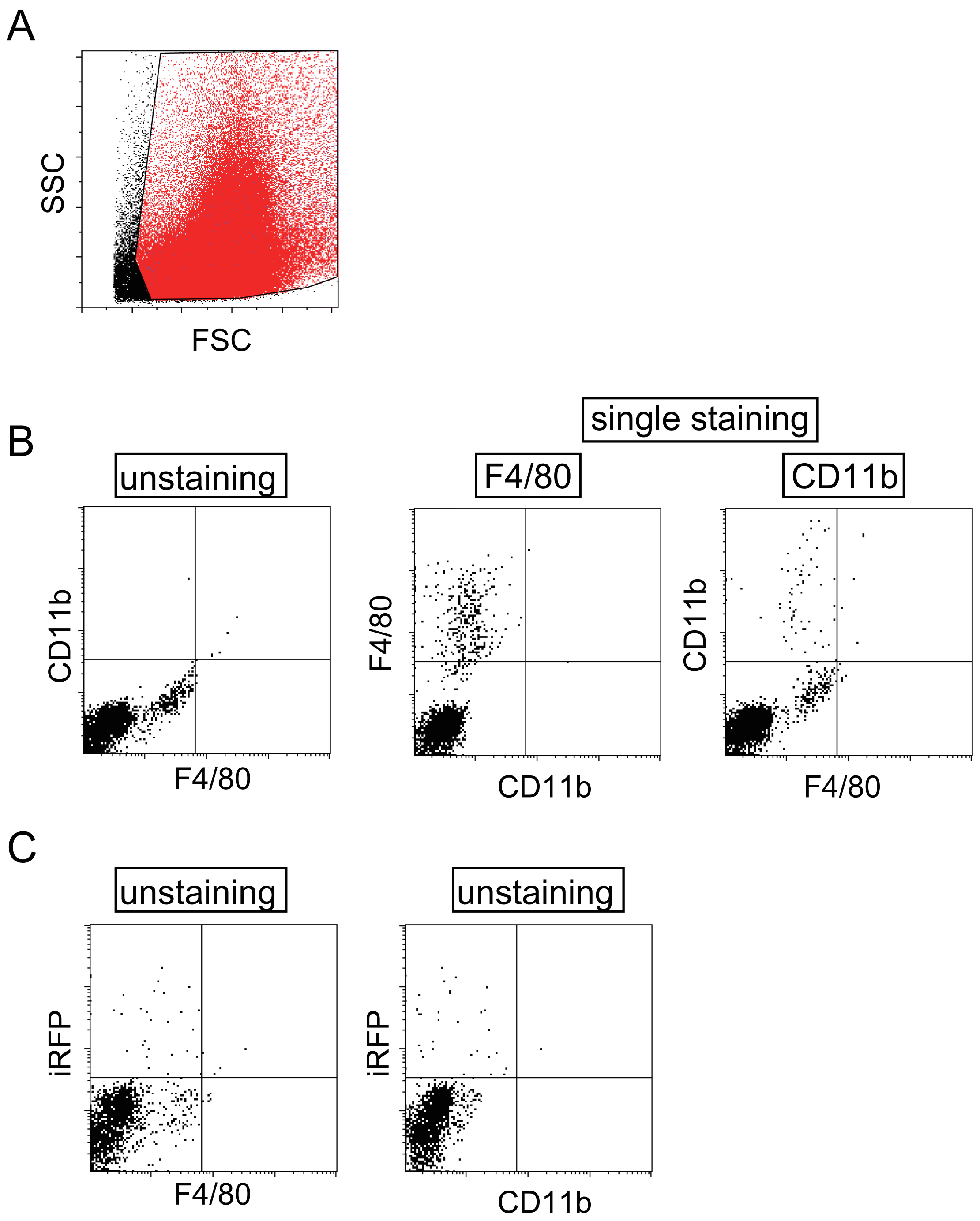

Supplementary Fig. 1 Gating strategy for FACS analysis

(A) Gating strategy for the data in Fig. 3C. (B) Wild-type compensation control for the data in Fig. 3C. (C) Unstaining compensation control of iRFP TG for the data in Fig. 3C.
